# The Role of Nitric Oxide, ADMA, and Homocysteine in The Etiopathogenesis of Preeclampsia—Review

**DOI:** 10.3390/ijms20112757

**Published:** 2019-06-05

**Authors:** Weronika Dymara-Konopka, Marzena Laskowska

**Affiliations:** Department of Obstetrics and Perinatology, Medical University of Lublin, Poland, 20-950 Lublin, Jaczewskiego 8, Poland; weronika.dymara@gmail.com

**Keywords:** preeclampsia, asymmetric dimethylarginine, nitric oxide, homocysteine

## Abstract

Preeclampsia is a serious, pregnancy-specific, multi-organ disease process of compound aetiology. It affects 3–6% of expecting mothers worldwide and it persists as a leading cause of maternal and foetal morbidity and mortality. In fact, hallmark features of preeclampsia (PE) result from vessel involvement and demonstrate maternal endothelium as a target tissue. Growing evidence suggests that chronic placental hypoperfusion triggers the production and release of certain agents that are responsible for endothelial activation and injury. In this review, we will present the latest findings on the role of nitric oxide, asymmetric dimethylarginine (ADMA), and homocysteine in the etiopathogenesis of preeclampsia and their possible clinical implications.

## 1. Preeclampsia—Background

Preeclampsia (PE) is a serious, pregnancy-specific, multi-organ disease process of compound aetiology. It affects 3–6% of expecting mothers worldwide and it persists as a leading cause of maternal and foetal morbidity and mortality [1]. The diagnosis of PE is clinical. The diagnostic criteria were revised in 2013 and 2014: it is defined as new onset hypertension developing after 20 weeks of gestation and the coexistence of a minimum of one of the following new onset conditions: proteinuria, maternal end- organ dysfunction (including renal, hepatic, haematological, or neurological complications), or uteroplacental dysfunction reflected in foetal growth restriction (FGR) [2,3]. The disease can be further clinically classified as PE with or without severe features, as well as early-onset syndrome (presenting before 34 weeks of gestation, versus late-onset after completed 34 weeks), preterm PE (occurring from 34 + 1 but before 37 + 0 weeks), and term PE (after completed 37 weeks) [4]. The current management of the disease mainly depends on gestational age and assessment of PE severity, focusing on blood pressure control, maternal and foetal surveillance, and it aims to deliver the baby in optimal condition prolongating the pregnancy without worsening state of the mother [3]. It requires individualized calculations of risks and benefits, but unfortunately delivery still remains the only definitive treatment.

In recent times, a huge progress has been made in understanding the disease, getting scientist and doctors closer to explain biological mechanisms underlying the development of PE that possibly can be used to create new therapeutic strategies targeting them.

**Figure 1:**
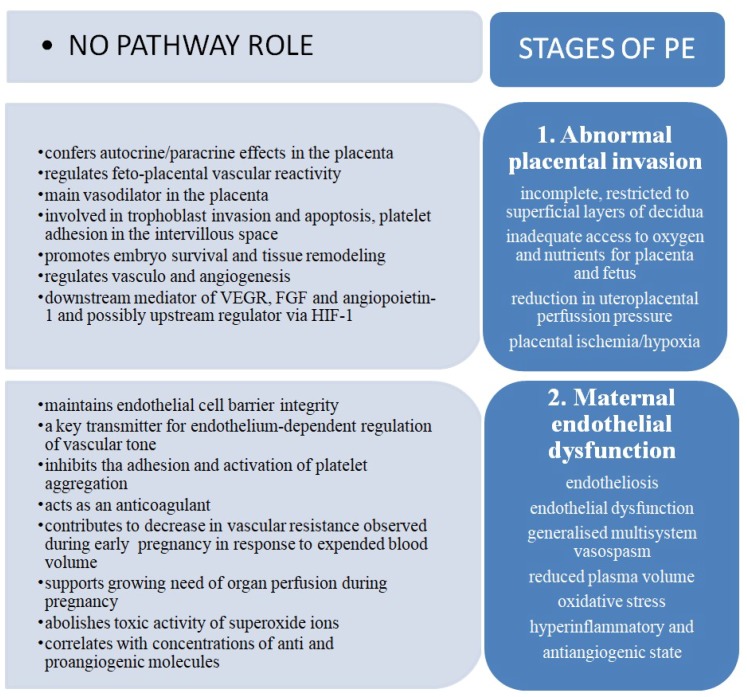
Physiological roles of NO pathway in pregnancy and their possible influence on preeclampsia (PE) development in two-stage model of disease. NO PATHWAY ROLE: Nitric oxide (NO) pathway role; STAGES OF PE: Stages of preeclampsia (PE).

Within the last decade, subsequent studies confirmed the hypothesis of Roberts and colleagues from 1989, who suggested that PE clinical manifestations might be due to maternal endothelium dysfunction [5]. In fact, the hallmark features of PE result from vessel involvement and demonstrate maternal endothelium as a target tissue. However, the placenta, as the interface between mother and fetus, is also regarded a key and causative player in pathogenesis of PE. Growing evidence suggests that chronic placental hypoperfusion triggers the production and release of certain agents that are responsible for endothelial activation and injury. (Figure 1)

The development of PE involves a two-stage process [6]. The first, crucially important step is asymptomatic and it takes place during placental invasion and differentiation. While, during normal placentation, the embryo-derived cytotrophoblast properly invades the uterine wall, including the myometrium and spiral arterioles and it leads to transformation of maternal spiral arteries into large capacitance and low resistance vessels; this process is defective in preeclampsia [7,8,9]. The invasion of cytotrophoblast is incomplete, restricted to superficial layers of decidua that provides inadequate access to maternal oxygen and nutrients for the placenta and growing foetus. Poor placental invasion leads to diminished uteroplacental perfusion pressure and ischemia.

Abnormalities of placental invasion anticipate maternal disorder. Clinical manifestations that define PE represent the second stage of disease. Chronic placental hypoperfusion triggers abnormal production and the release of numerous bioactive factors into the maternal circulation. These circulating substances target endothelial cells resulting in widespread endotheliosis, endothelial dysfunction, generalized multi-system vasospasm, reduced plasma volume, oxidative stress, and hyperinflammatory state. Excessive expression of antiangiogenic proteins, like soluble fms-like tyrosine kinase 1 (sFlt-1) and soluble Endoglin (sEng), which catch circulating, decreased proangiogenic substances, like vascular endothelial growth factor (VEGF), placental growth factor (PlGF), and transforming growth factor β (TGFβ) result in an understanding of PE as an antiangiogenic state [10,11,12,13].

Defective trophoblast invasion is an early event in preeclampsia development. However, it has not been resolved whether it is the reason or result of another underlying problem. It remains unclear why trophoblast invasion is interrupted, but an altered immunological response at maternal-foetal interphase, genetics, and environmental factors are believed to contribute, although their role may vary between patients [14,15]. Furthermore, it is suggested that maternal susceptibility and response to placental derangements determines the onset, severity, clinical manifestations, and progression of the disease [16]. The most recent theory identifies PE as a complex disease with two distinct clinical presentations. The first, placental phenotype is associated with shallow trophoblastic invasion and restricted foetal growth, as opposed to PE associated with maternal metabolic syndrome. The second phenotype is associated with normal fetal growth and maternal low-grade inflammation, mainly due to placental oxidative stress, placental villi overcrowding, and decidual lesions [17,18].

Nitric oxide (NO) is one of the key players in the regulation of placental blood flow. It is actively engaged in cytotrophoblast endovascular invasion and development of the placenta, through its unique angiogenic and vasculogenic properties [19]. Current evidence supports altered NO production in the feto-placental unit in preeclampsia, which, by reduced bioavailability, may contribute to vasoconstriction of the placental bed, abnormal placental perfusion, and its maternal consequences, like increased blood pressure, systemic vascular resistance, and sensitivity to the pressors [20,21,22,23,24,25].

Asymmetric dimethylarginine (ADMA), which is an endogenous inhibitor of NO synthase (NOS), has also been associated with impaired endothelial function and with uterine artery flow disturbances that are characteristic for preeclampsia [24,26,27].

Homocysteine (Hcy) elevated concentrations in preeclamptic women lead to elevated ADMA levels, since Hcy has an inhibitory effect on ADMA metabolism. Hyperhomocysteinemia (HHcy) is also associated with endothelial cells lesions due to vascular fibrosis, which results in alterations in the coagulation system, enhanced platelet activation, and thrombogenesis—changes that are noted in preeclampsia [28,29,30].

In this review, we will present the latest findings on the role of nitric oxide, ADMA, and homocysteine in the etiopathogenesis of preeclampsia and their possible clinical implications.

## 2. Metabolism and Biological Role of NO, ADMA, and Homocysteine

Furchgott initially described nitric oxide as an endothelium-derived relaxant factor (EDRF) in 1980 after attributing a vasodilatory effect on vascular smooth muscle by stimulation of cholinergic nerves to the endothelium [31]. The identification of EDRF as NO was reported seven years later and was awarded a Nobel Prize in Physiology or Medicine for Furchgott, Ignarro, and Murad in 1998 for their discoveries concerning nitric oxide as key transmitter in the cardiovascular system. NO is produced through L-arginine-NO synthase pathway by converting L-arginine to L-citrulline in the presence of oxygen and the cofactor tetrahydrobiopterin or alternative enzymatic and non-enzymatic nitrate-nitrite-NO pathways [32,33].

Nitric oxide synthase (NOS) possess three different isoforms, namely neuronal NOS (nNOS) or type 1, inducible NOS (iNOS) or type 2 and endothelial NOS (eNOS) or type 3 [32]. NOS1 and NOS3 are considered as constitutive NOS. Endothelial NOS is stored in plasma membrane caveolae and its distribution and activity are regulated by numerous mechanisms [34]. Being released from endothelial cells, NO is quickly transported to the closest vascular smooth muscle cells, where it exerts its role by inducing the production of cyclic guanosine monophosphate (cGMP) as a second messenger. It may be neutralized by reactive oxygen species on the way to its target cells [35].

NO is the key transmitter for the endothelium-dependent regulation of the vascular tone that is controlled by humoral, metabolic and mechanical factors, for example, in response to increased blood flow [36]. Furthermore, NO inhibits the adhesion and activation of platelet aggregation, abolishes the toxic activity of superoxide ions, and acts as an anticoagulant and antiatherogenic substance [22,32].

It is also considered to have major effects on the gestational endothelial function as well as to play a supportive role in promoting embryo survival, tissue remodelling, immunosuppression, and vasoregulation critical for placental nutrient transport [37,38,39]. The human foeto-placental vasculature lacks autonomic innervation and, therefore, NO confers autocrine and/or paracrine effects, influencing different aspects of physiological pregnancy. In particular, NO is the main vasodilator that is involved in foeto-placental vascular reactivity regulation, placental bed vascular resistance, trophoblast invasion and apoptosis, and platelet adhesion and aggregation in the intervillous space [40].

Further, the role of NO is also established in vasculogenesis, which results from the de novo formation of vessels derived from pluripotent precursor cells and angiogenesis, the formation of functional capillaries from pre-existing vasculature. Vascular endothelial growth factor (VEGF) is a key particle in these processes. Its expression is mediated by NO release and was required for initiation of vasculogenesis [41]. NO is also a critical downstream mediator of other than VEGF potent angiogenic substances, like basic fibroblast growth factor (FGF), and angiopoietin-1 [42]. The critical role of NO in angiogenesis has been shown in eNOS knockout mice [43]. NOS inhibition is accompanied by defective angiogenesis, as exemplified by deficient vascular sprouting. Interestingly, NO may also act upstream of angiogenic growth factors, because hypoxia-inducible factor-1 (HIF-1) perhaps mediated the effect of NO on VEGF production [44].

Asymmetric dimethylarginine (ADMA), which is an analogue of L-arginine, constitutes a natural metabolite that is found in human plasma. Dimethylarginines are formed as a result of the degradation of methylated arginine residues in proteins [45]. Approximately 80% of ADMA undergoes enzymatic transformation by two dimethylarginine dimethylaminohydrolases (DDAH-1 and -2) to L-citrulline and dimethylamine, whereas kidneys excrete the rest. ADMA is endogenous competitive inhibitor of L-arginine for all three isoforms of NOS. Elevated levels of ADMA block NO synthesis and limit the cellular uptake of L-arginine, thereby contributing to oxidative stress and disrupting further NO biogenesis. In this way, ADMA impairs the endothelial function and thus promotes atherosclerosis. Therefore, it is recognized as a biomarker of endothelial disorders. The ADMA levels are found to be elevated in patients with various cardiovascular and metabolic conditions, such as hypercholesterolemia, atherosclerosis, hypertension, chronic heart or renal failure, diabetes mellitus, stroke, and hyperhomocysteinemia [29,45,46,47]. ADMA has been shown to increase systemic vascular resistance in humans, as an endogenous inhibitor of NOS [45].

Homocysteine (Hcy) is a sulfur-containing amino acid that is produced during the conversion of essential amino acid methionine (Met) to cysteine (Cys) [48]. Its synthesis occurs in the transsulfuration of dietary methionine, which is abundant in animal protein, but it can also occur in demethylation that is related to fasting conditions. Hcy is metabolized by one of the two following pathways: remethylation to methionine, which requires the addition of a methyl group from 5-methyltetrahydrofolate (5-methyl THF) and the cofactor vitamin B12 (or betaine in an alternative reaction, restricted to the liver and independent of vitamin B12); and, transsulfuration to cystathionine; and finally, to cysteine, which requires vitamin B6 as a cofactor [49]. Methionine derivative, S-adenosyl methionine, is a cofactor that serves as a most important methyl donor of the body, whereas cysteine is used for glutathione synthesis or it is metabolised into taurine.

5-methyltetrahydrofolate (5-MTHF), which is the predominant circulating form of folate is the result of a reduction of 5,10-methylenetetrahydrofolate (5,10-MTHF) catalysed by the MTHFR (methylenetetrahydrofolate reductase) enzyme, coded by MTHFR gene, whose locus is on chromosome 1 at the end of the short arm (1p36.6) [50,51]. Polymorphisms of the MTHFR gene play a significant role in the pathogenesis of hyperhomocysteinemia.

The definition of hyperhomocysteinemia (HHcy), generally understood as increased homocysteine in the blood, differs between authors [52]. The total fasting concentration of Hcy in plasma of healthy patients is low and its level is 5.0–12.0 µmol/l when the immunoassay methods are used or between 5.0 and 15.0 µmol/L when assessed with the use of HPLC (high-performance liquid chromatography) [53]. Modarate HHcy is diagnosed if the levels are within the range of 16 to 30 µmol/L, 31–100 µmol/L is considered to be intermediate and a value above 100 µmol/L is classified as severe hyperhomocysteinemia [54].

The main causes of elevations in homocysteine levels are vitamin deficiency (B6,B12,folate), aforementioned genetic defects in enzymes that are involved in its metabolism (cystathionineβ-synthase deficiency and MTHFR), and disease conditions that interfere in the metabolism of cofactor levels, disturbing the transsulphuration and remethylation processes. In the general population, higher values of Hcy are observed in men then in women, although the discrepancy diminishes with age and in postmenopausal patients who tend to have higher Hcy levels [55].

In general, we can divide HHcys in two types: severe, but rare forms due to major genetic defects (individuals with the rare homocystinuria typically have levels of >100 μmol/L) and more common, moderately elevated homocysteine levels that are related to a pathogenesis, such as genetic and environmental factors, which is observed in up to 5% to 12% of the general population [52,56].

The most common cause of severe hyperhomocysteinemia and classic homocystinuria (congenital homocystinuria) is considered to be the homozygous deficiency of CβS (cystathionine-β-synthase). This defect is responsible for an increase as much as up to 40-fold in fasting total homocysteine. Other not often observed genetically conditioned states of HHcy are the homozygous deficiency of MTHFR, deficiency of methionine synthase, and impaired activity of methionine synthase due to impaired vitamin B12 metabolism [54].

However, the most common genetic deficiency, which occurs at large rates in various populations, is single nucleotide polymorphism of MTHFR that has been associated with mild and moderate (25–60 μmol/L) hyperhomocysteinemia [57]. A point mutation C-to-Tsubstitution at nucleotide 677 (677C→T) in the gene for MTHFR causes a thermolabile variant of the enzyme and has half-reduced activity, whereas in people who are homozygousfor MTHFR C677T, there is only 30% of normal enzyme function [54]. Another point mutation, called MTHFR A1298C, leads to 60% of normal enzyme function. Double heterozygous (1 abnormal MTHFR C677T gene plus 1 abnormal MTHFR A1298C gene) results in decreased reductase activity as those homozygous for the C677Tpolymorphism [58].

However, the leading cause of HHcy is folate, vitamin B12, and less commonly, B6 deficiency due to low supply, malabsorption, and treatment with substances, such as cyclosporin, methotrexate, fibrates, Levodopa (L-DOPA), and carbamazepine that interfere with the metabolic paths of these vitamins [59,60]. High Hcy levels have been also associated with impaired renal function, high plasma creatinine, smoking, coffee consumption, and alcoholism [52].

HHcy is generally recognized as an independent risk factor for coronary, cerebral, and peripheral atherosclerosis, which was first reported by McCully in 1969 and later confirmed in a meta-analysis of numerous additional studies [61,62,63]. An extend meta-analysis suggested that an increment of homocysteine of 5 mmol/L is comparable to the increase in the risk of coronary artery disease caused by cholesterol elevation of 0.5 mmol/L [62]. An association between HHcys and cardiovascular disease, as well as some age-related pathologies, like stroke, Alzheimer’s disease, Parkinson’s disease, chronic renal failure, and osteoporosis is widely described [61,62,63,64,65,66,67,68,69,70,71,72]. There are ongoing efforts to understand if HHcy observed in vascular diseases is a causative factor or a consequence of endothelial activation [73].

## 3. NO, ADMA and Homocysteine in Pregnancy

During uncomplicated pregnancy, increased NOS activity in human uterine artery leads to higher NO levels [74]. NOS3 expression raises primarily in the syncytiotrophoblasts and NOS2 activity grows throughout pregnancy, with a peak around mid-gestation [38,39,75,76]. Physiological reduction of blood pressure during pregnancy may greatly rely on the vasodilatory action of NO. NO contributes to the vasodilatation of blood vessels and the decrease in vascular resistance observed during early pregnancy, when maternal blood volume expands, while systemic vascular resistance and systemic blood pressure both decline [22,25,75,76,77].

In normal pregnancy, also levels of cGMP, a second messenger of NO signalling is particularly increased during the first trimester in plasma and urine [78]. Furthermore, a NO-cGMP pathway is present in the human uterus and it may be responsible for maintaining its relaxation. Spontaneous contractility in vitro was enhanced by the NOS inhibitor L-NAME (nitro-L-arginine methyl ester) and decreased by NO. Thus, uterine reduced the responsiveness to nitric oxide at term may play a role in the initiation of labour [79].

Different studies on total NO in pregnancy gave conflicting results. The measurement of its relatively stable metabolites, nitrate, and nitrite (NOx) is often employed as an indicator of NO production and as a marker of NOS enzyme activity because NO is highly labile molecule [80]. Still, the plasma level is influenced, not only by the production, but also by the clearance of NO derivatives [79]. Some studies found that NO production increases with gestational age during normal pregnancy, especially in the second trimester, and it peaks in the third trimester [81,82,83]. However, contrary results were also published reporting that maternal circulating nitrite level decreased with advancing gestation [84], or even that there were no changes in NO production when compared to the nonpregnant state [85,86].

Likewise, studies investigating the circulating levels of NO in preeclampsia have also reported conflicting results [87]. These observations suggest that the status of NO biosynthesis in women during normal pregnancy and preeclampsia remains to be defined.

### 3.1. ADMA in Pregnancy

In normotensive pregnancy, the maternal plasma ADMA levels are generally reduced when comparing to non-pregnant group. The lowest concentration of ADMA is described during the first trimester, when the early fall in blood pressure is accompanied by a significant fall in ADMA concentration. The ADMA levels increase with gestational age in the second and third trimester [24,26]. These findings lead to a conclusion that, in early pregnancy, the reduction in ADMA and concomitant increase in NO are responsible for previously described hemodynamic adaptation, a higher need of organ perfusion in pregnancy, and uterine relaxation. In advanced pregnancy, physiologically increased ADMA levels thus help to prepare the uterine muscle fibers for the higher contractile activity before the labour. This is reflected by the higher ADMA concentrations after caesarean birth when compared with vaginal delivery and it may contribute to decreased nitric oxide production and bioavailability in neonatal vascular beds [88].

### 3.2. Homocysteine and Pregnancy

The homocysteine plasma levels fall in normal pregnancy [89,90]. An increase in plasma volume and associated haemodilution, glomerular hyperfiltration and postulated raised foetal need for methionine are mechanisms considered to contribute to this effect [91]. The importance of homocysteine to early foetal metabolism is demonstrated in a number of studies [92].

The reference values for HHcys in pregnancy that are proposed in one study were established as: higher than 7.7 mmol/L in the second trimester, and 10.5 mmol/L in the third trimester [93], although different authors usually defined their own cut-off values.

In the pathology of pregnancy, the disturbance of maternal homocysteine metabolism has been linked with recurrent pregnancy loss, deep venous thrombosis, foetal neural tube defects, and various conditions characterized by placental vasculopathy, such as preeclampsia, foetal growth restriction, and abruption [94,95,96,97,98].

## 4. NO, ADMA, and Homocysteine in Preeclampsia

### 4.1. NO Pathway Dysfunction in PE

In preeclamptic patients, like in normotensive pregnancies, the measurements of total NO concentration have shown variable results, ranging from decreased [81,99,100], unchanged [101], and increased [102,103] levels of circulating NO metabolites. The dietary intake of these substances could also influence the disparity, although a study that was conducted on women with PE, subjected to a reduced nitrate/nitrite diet, did not show decreased endogenous NO production [78]. In fact, NO measurements may be difficult to interpret, since they reflect the total activity of all three isoforms of NOS, not just endothelial. While the whole body NO may not change in PE, in view of the evidence for reduced endothelial NO signalling and decrease in vascular relaxation in PE, tissue-specific differences in NOS expression and NO bioavailability could be expected. For instance, in late pregnant rats, renal eNOS decreases by 39%, while iNOS and nNOS increase by 31% and 25%, respectively [104].

PE is associated with abnormalities eNOS-NO pathway that probably exists at different stages of signal transduction process. There is not one particular defect, but multiple changes in key regulatory aspects in NO signaling. In studies where serum from PE women was placed on isolated vessels, nitric oxide-mediated vasorelaxation appeared to be absent [105].

Some studies have indicated that measuring plasma nitrite levels may reflect endogenous NO formation because NO is rapidly oxidized to nitrite. This is because 70% of plasma nitrites derive from NO synthase activity in the endothelium and its inhibition was associated with corresponding decreases in plasma nitrite concentrations [106,107]. By only using nitrite levels (which may be a better measure than total nitrite + nitrate), a reduction from 40 to 60% of total whole blood or plasma nitrite concentration was reported in PE women [108,109,110].

There is clinical evidence for the link between impaired NO formation and antiangiogenic factors overexpression in preeclampsia. Significant negative correlation between two antiangiogenic factors: sEng and sFlt-1, and nitrite concentrations was described [110], which suggested a possible inhibitory effect caused by these substances on the production of NO in patients with preeclampsia. Experimental studies have shown that NO increases proangiogenic VEGF and PIGF and it decreases sFlt-1 in hypoxic human trophoblast cells [111].

Using the nitrate reductase assay to measure NO, also a correlation between reductions in plasma NO with disease severity was identified, such that the levels were about 30% lower in severe PE vs. healthy pregnant controls [112].

Attempts to assess eNOS activity in PE led to the conclusion that it is still unknown whether eNOS deficiency plays a causal role there. In the murine model, chronic NOS inhibition reversed systemic vasodilation and glomerular hyperfiltration in pregnancy, which suggested its role for endothelial damage and decreased NO in the pathogenesis of preeclampsia [113]. However, different study with the use of eNOS knockout mice showed reduced uterine artery diameter, spiral artery length, and, as a consequence, diminished uteroplacental blood flow, resulting in elevated markers of placental hypoxia in the junctional zone. Even so, interestingly, sFlt-1concentration was not elevated in the eNOS knockout mice [43].

Data from PE women is quite limited and without consensus on eNOS expression, as higher, lower, and unchanged levels of mRNA or enzyme have been reported. Several human studies failed to detect any significant differences in the circulating levels of eNOS [114,115]. One of the earliest studies on eNOS activity found an increase in eNOS expression in syncytiotrophoblast, foetal terminal villous capillary, and stem villous vessel endothelium, whereas the lack of eNOS expression in vascular terminal villi and weak expression in endothelial cells of villous vessels in placenta from normal pregnancy was noted [116]. These results are supported with a recent finding that caveolar eNOS expression is increased in PE placentas [117]. However, this stays in contrast to the observed similar placental levels of eNOS activity in PE patients [118], and to a more recent study in which placental syncytiotrophoblast eNOS expression was even decreased [119]. Apart from these confusing findings, altered placental eNOS levels may not directly relate to peripheral vascular endothelial function.

The reduced availability of eNOS substrate (L-Arginine) or the competitive inhibition by ADMA constitute other factors that may contribute to dysfunctional endothelial NO signalling in PE. One of proposed animal PE models involves administering the aforementioned L-NAME, a competitive inhibitor of arginine, which leads to maternal hypertension and proteinuria, and reduced foetal weight in a dose-dependent way [120]. L-NAME-induced hypertension and high circulating levels of sFlt-1 could be attenuated by the administration of exogenous sodium nitrite, and in this way restoring NO bioavailability [121].

In pregnancies that are complicated by PE, the ADMA levels are significantly higher than in both normotensive gestational age-matched and the nonpregnant control group [24,115,122,123,124]. Even higher concentration of ADMA in patients with early-onset PE may suggest a relationship between disease severity and determining the time of PE clinical manifestation [125,126]. Moreover, ADMA may have a predictive value in PE, since its modestly (+26%) elevated concentrations were observed as early as in the first trimester [127] and significantly elevated during second trimester in pregnancies that developed PE in more advanced gestational age [128]. Another hypotesis is that increased ADMA concentration may contribute to development of PE in early pregnancy, leading to impaired placentation and its consequences [126]. The association of abnormal uterine artery Doppler waveforms with elevated ADMA levels [129,130] supports the role of endogenous NOS inhibitors adversely affecting maternal vasodilation and blood pressure.

It has been postulated that hyperhomocysteinemia (HHcy) may contribute to the development of PE, as it leads to endothelial dysfunction and accumulation af ADMA [29,30].

### 4.2. Homocysteine in PE

The association of hyperhomocysteinemia and preeclampsia has initially been suggested by Decker et al. [131], and all authors have not confirmed it. However, the majority of evidence suggests a positive correlation.

Higher maternal plasma homocysteine concentrations in preeclamptic pregnancies as compared to normotensive were widely reported [93,125,130,132,133,134,135,136,137]. Overall, these differences seem to be present at all of the investigated time points across the gestation, starting from early pregnancy before 20 weeks [138,139,140,141,142] and in both severe and non-severe forms of PE. Comprehensively, this finding led to a conclusion that high homocysteine in early pregnancy constitutes a risk factor for PE. Therefore, attempts to introduce Hcy measurement into a screening test for PE to improve the prediction model, for example, by combining it with uterine artery Doppler test in the second trimester, resulted in being valuable [140]. However, there are also studies that rejected an association between elevated Hcy in early second trimester and subsequent PE [143,144,145]. This could result from differences in study designs, laboratory techniques, and disease definitions, among other reasons, which may be further investigated in a systematic review.

Still, most of the papers focus on the third trimester [93,130,132,133,134,135,137], where the differences between HHcys in severe and non-severe PE are marked. Pregnant women complicated with severe preeclampsia displayed significantly higher serum Hcy levels than with non-severe form [93,112,132,134,136,146]. Thus, homocysteine concentrations positively correlating with the clinical presentation of disease may constitute a marker of severity of preeclampsia.

Besides elevated Hcy concentrations in maternal plasma, the majority of authors analyse the possible association with either folate or vitamin B12 deficiencies, as well as NO pathways in preeclamptic patients. Here, contrary results are presented. Folate and vitamin B12 serum levels were described as unchanged [132,134,135] in PE when comparing to normotensive healthy women with uncomplicated pregnancies. However, opposite results indicating these vitamins deficiencies were also reported [135,146]. Nevertheless, further studies are needed to confirm whether the prescription of these vitamins could decrease serum homocysteine, thereby possibly reducing the risk of preeclampsia or (if it occurs) its severity.

Interesting association between HHcy and NO signalling pathway was reported. Homocysteine inhibits the expression and activity of dimethylamino dimethyl hydrolase (DDAH), which is the enzyme degrading ADMA to citrulline and dimethylamine [22,147,148,149]. It has been suggested that elevated ADMA is a mediator of endothelial dysfunction in hyperhomocysteinemia due to this metabolic relation [148,150]. HHcy, leading to accumulation of ADMA, may contribute to eNOS blockade and NO deficiency in the presence of general appropriate concentration of its synthase. The reduced release of NO by endothelial cells in HHcy was observed, suggesting the impairment of the eNOS pathway by DDAH inhibition [29].

The hypothesized mechanisms of disease linking homocysteine to preeclampsia are complex and still incompletely understood.

To date, vascular endothelial cell dysfunction that is provoked by an elevated level of homocysteine (Hcy) is suggested to be the most important connection. However, some authors questioned whether mild HHcy observed in PE, with Hcy values that are similar to those found in normotensive non-pregnant women, can provoke damage of the vascular endothelium. They postulate that this damage can be mediated rather by oxidative stress, as endothelium of pregnant women might be more vulnerable to oxidative injury [151]. Hyperhomocysteinemia is also associated with lesions in endothelial cells, due to vascular fibrosis, which results in alterations in coagulation system, enhanced platelet activation, and thrombogenesis—changes that are noted in preeclampsia [28,29,30].

On the other hand, metabolism in the kidney is the major route by which homocysteine is cleared from plasma. The association between Hcy and glomerular filtration rate (GFR) seems linear and it is present, even in the hyperfiltrating range [152,153]. Thus, this route of elimination may be affected by the already established preeclamptic changes in the kidney and secondarily lead to increased Hcy concentrations in plasma [154].

## 5. Therapeutic Potential of NO Pathway during Pregnancy

Enhancing the NO signalling in pregnancy is still thought to be an attractive option in both preeclampsia prevention in high-risk groups of women and treatment. There are attempts to prolong the pregnancy with the use of NOS substrates (L-arginine and L-citrulline), as well as NO-donors (glyceryl trinitrate, S-nitrosoglutathione, isosorbide mononitrate), natural derivatives, or vasodilators, such as sildenafil citrate [155,156,157,158]. NO donors and substrates have been also used for the management of other pregnancy disorders, like recurrent abortions, treatment of preterm labour, and dysmenorrhea [155]. Unfortunately, the analysis of use of these substances in PE gives conflicting results.

In vitro, nitric oxide synthase activity is inhibited by intracellular ADMA and is rescued by L-arginine [159]. Therefore, L-arginine supplementation in pregnancy could possibly overcome NOS blockage and its consequences. A study of intravenous infusion of l-arginine in pregnant women showed a significant reduction in blood pressure—an effect that was greater in women with preeclampsia [157,160]. Additionally, a significant reduction in PE incidence was described in high risk women who received L-arginine and vitamins C and E (as antioxidants reducing oxidative stress implicated in the pathophysiology of PE) before 24 weeks of gestation, when comparing to placebo and only vitamin group, although the effects of L-arginine alone were not studied [161]. However, last year, meta-analysis showed that combined vitamin C and E supplementation has no influences on the occurrence of preeclampsia [162], although an interaction between these vitamins and L-arginine is not well explained. Supplementation of L-arginine for pregnant women with chronic hypertension showed less need for antihypertensive drugs use, but no reduction in the incidence of superimposed preeclampsia [163]. Interestingly, women who go on to develop PE have been found to have higher, not lower, plasma L-arginine concentrations [129], which can counteract the raised ADMA values, but other vasoconstrictor effects may persist in women who are vulnerable to preeclampsia.

The Cochrane database systematic review, including six trials demonstrates, that there is insufficient evidence to draw reliable conclusions about whether NO donors and precursors prevent PE or its complications. Another 13 papers are still waiting to be assessed [164]. Conclusions from the review are limited mainly due to the fact that too few women have been studied. Adverse effects that were described during supplementation of NO donors included mainly headaches, often sufficiently severe to stop medication. However, more recent studies clearly show that isosorbid mononitrate and L-arginine are both effective in prevention of PE [165,166]. Besides the significant reduction of PE incidence in the treatment groups, there was also a reduction in FGR and improvement in foetal outcome, including less neonatal admissions to the intensive care unit.

An inorganic nitrate, NO3, with a capacity to increase the bioavailability of NO has been recently implemented in a clinical trial with beetroot juice as a norganic nitrate (NO3^−^) rich dietary supplement [167]. It was the first trial to investigate effects of nitrate supplementation on blood pressure in human pregnancy, according to the authors. The treatment group consisted of pregnant women with chronic hypertension. In this group, the administration of NO3^−^ donors significantly increased plasma and salivary nitrate/nitrite when compared with placebo, but there was no overall reduction in blood pressure. However, there was a highly significant correlation between changes in plasma nitrite and only lowering diastolic blood pressure in the nitrate-treated arm [168]

Sildenafil exerts its role by inducing vasodilatation, via inhibiting phosphodiesterase and thereby maintaining the availability of cGMP, the effector of NO activity within the cell [169]. In vivo studies of sildenafil citrate in rat models of preeclampsia have shown a significant reduction in the production of antiangiogenic molecules sFlt-1 and sEng [170], as well as an improvement in blood pressure, proteinuria, uteroplacental and foetal perfusion after treatment [171]. Lately, a randomized controlled trial to evaluate PE therapy with sildenafil showed that, when compared to controls (receiving a placebo), therapy with sildenafil resulted in four days prolongation of pregnancy [158].

Glyceryl trinitrate (GTN), which is commonly known as nitroglycerin, is a widely used organic nitrate in clinical practice, particularly for the treatment of angina pectoris. Transdermal nitroglycerin patches have been the focus of various studies, for both the prevention and management of PE and related disorders. Studies of both forms: transdermal [172,173] and sublingual GTN [156] in preeclamptic patients consistently showed a significant reduction in blood pressure and resistance in the uterine artery without an adverse effect on the foetal Doppler parameters. Nonetheless, these studies have highlighted the potential use of GTN as an antihypertensive agent in PE. However, it remains to be established whether GTN offers any competitive advantage over already existing treatment options. Certainly, the major disadvantage of organic nitrates, in general, and GTN, in particular, is the development of tolerance upon continuous dosing, necessitating the requirement of regular ‘nitrate-free’ intervals.

## 6. Conclusions

New therapeutic options emerge as our understanding of preeclampsia improves. Enhancing NO pathway, by overcoming eNOS block and neutralizing raised ADMA values and by homocysteine reduction, may be a perspective goal in the treatment and prevention of PE.

Still new, prospective clinical trials are needed to safely develop effective management strategies with the use of pharmacological modulators of the NO system, which seem to hold promise for the treatment of preeclampsia.
